# Exploring the Potential of Olive By-Products in Bísaro Pig Feed: Effects on the Chemical Compositions and Fatty Acid Profiles of Three Different Muscles

**DOI:** 10.3390/foods14050836

**Published:** 2025-02-28

**Authors:** Jessica Paié-Ribeiro, Victor Pinheiro, Cristina Guedes, Maria José Gomes, José Teixeira, Ana Leite, Lia Vasconcelos, Alfredo Teixeira, Divanildo Outor-Monteiro

**Affiliations:** 1 Animal Science Department, University of Trás-os-Montes and Alto Douro (UTAD), 5000-801 Vila Real, Portugal; vpinheir@utad.pt (V.P.); cguedes@utad.pt (C.G.); mjmg@utad.pt (M.J.G.); joseteixeira@utad.pt (J.T.); divanildo@utad.pt (D.O.-M.); 2 Veterinary and Animal Research Centre (CECAV), University of Trás-os-Montes and Alto Douro, 5000-801 Vila Real, Portugal; 3 AL4AnimalS, Quinta de Prados, 5000-801 Vila Real, Portugal; 4 Mountain Research Center (CIMO), Polytechnic Instituto of Bragança, Campus de Santa Apolónia, 5300-253 Bragança, Portugal; anaisabel.leite@ipb.pt (A.L.); lia.vasconcelos@ipb.pt (L.V.); teixeira@ipb.pt (A.T.)

**Keywords:** olive by-products, exhausted olive cake, meat quality, Bísaro breed, circular economy

## Abstract

The olive oil industry generates large quantities of olive cake (OC), making its use in animal feed an environmentally sustainable alternative. Considering that the ham of Bísaro pigs is traditionally used for the production of cured ham, the analysis of raw meat is essential to understand how diet influences its quality before the maturation process. This study examined the effect of different types of OC in the diets of Bísaro pigs, focusing on the chemical compositions and fatty acid profiles of three fresh ham muscles—*biceps femoris* (BF), *semimembranosus* (SM), and *semitendinosus* (ST). Forty Bísaro pigs were assigned to five diets: control (CD), 10% raw OC (COC), two-phase OC (TPOC), exhausted OC (EOC), and exhausted OC with 1% olive oil (EOC-OO). The diets significantly influenced moisture, protein, collagen, and haem pigments (*p* < 0.05). TPOC had the highest moisture content, while CD had the lowest. Protein levels were higher in BF and SM for OC-fed pigs. Collagen in ST was highest in CD and lowest in EOC. Haem pigments varied, with EOC highest in BF and ST and EOC-OO highest in SM. Significant MUFA differences were found in BF (*p* < 0.001), highest in CD and lowest in TPOC. PUFA levels and the PUFA/SFA ratio were highest in TPOC (*p* < 0.001), while SFA remained unchanged. The results suggest that up to 10% OC can be included in pig diets without compromising meat quality, but factors such as economic viability and nutritional variability must be considered. This study provides new insights into how OC affects muscle composition, contributing to optimizing feeding strategies for better meat quality and sustainability.

## 1. Introduction

The rapid growth of the global population, coupled with the rise in per capita income in developing countries, is driving an increased demand for food. Furthermore, shifting consumer habits have led to a significant rise in the demand for animal products [1,2]. According to the FAO (Food and Agriculture Organization of the United Nations), it is projected that by 2050, more than half of the world’s population will live in urban areas, necessitating a doubling of food production to meet this demand [2]. Global meat consumption is projected to grow at an annual rate of 2%, reflecting the increasing demand for animal protein. Over the forecast period, the average per capita meat demand is expected to rise by 2% from the 2020–2022 baseline to 2032, posing a significant challenge in ensuring sustainable production to meet this growing demand [3].

Amid these trends, agricultural waste reuse and sustainable feed alternatives have gained increasing attention due to market volatility in traditional feed ingredients and the growing volume of agro-industrial waste [4,5]. Incorporating by-products into animal feed has emerged as a promising solution to reduce the environmental impact and promote a circular economy based on a zero-waste approach. These by-products serve as valuable raw materials for animal nutrition [6].

Olive cake (OC), a by-product of olive oil extraction, presents a significant environmental challenge due to its complex storage and disposal requirements, as it can be highly toxic [7]. However, incorporating OC into animal feed offers multiple benefits, particularly in enhancing meat’s chemical, physical, and sensory properties, owing to its high content of antioxidants and bioactive phenolic compounds [8,9,10]. Moreover, OC has a considerable residual oil content, making it a promising alternative to improve the lipid profile of animal diets [11].

Among the olive oil industry’s by-products, olive leaves and OC are the most commonly used in animal feed. OC consists of pulp, stone, skin, and water, with mechanical stone removal yielding additional by-products such as olive pulp and olive pomace. On average, 40 kg of pomace is generated per 100 kg of processed olives [12].

The nutritional composition of pork is influenced by intrinsic and extrinsic factors, including breed, genotype, sex, age, production system, and diet [13,14]. These factors affect key meat attributes, such as (1) physical properties (weight, color, and intramuscular fat content); (2) carcass yield (weight, external fat thickness, and lean-to-fat ratio); (3) sensory quality; and (4) chemical composition (fat content, fatty acid profile, protein, moisture, vitamins, haem pigments, and more) [14,15].

The Bísaro pig, one of Portugal’s three native breeds, is primarily reared in the northeastern Trás-os-Montes region and is renowned for its high-quality meat. Its production plays a crucial role in preserving the breed and promoting traditional charcuterie, with some products classified under Protected Designation of Origin (PDO) and Protected Geographical Indication (PGI) [16].

This study explores the integration of OC as a feed ingredient for native Bísaro pigs, aligning with circular economy principles and sustainable production systems. Using OC in pig feed can potentially add value to agro-industrial waste, optimize resource use, and enhance environmental sustainability [17].

Fresh pork muscle’s chemical and fatty acid composition is a crucial indicator of meat quality, nutritional value, and safety. Fresh muscle tissue provides an unaltered profile of proteins, lipids, and fatty acids, allowing for an accurate assessment before post-mortem alterations occur [18]. Studies show that dietary interventions significantly impact pork’s fatty acid composition, with variations across different muscles due to intramuscular fat content and feed composition [19]. The inclusion of olive by-products such as olive stone and olive oil in pig diets has been shown to enhance the lipid profile by increasing MUFA and PUFA, both of which are associated with improved health benefits [10,11,20,21].

In this context, analyzing fresh muscle composition provides valuable insights for improving pork quality, optimizing production, and enhancing consumer satisfaction. A deeper understanding of muscle fiber characteristics, combined with targeted nutritional strategies, supports the development of nutritionally superior pork products, aligning with increasing market demands [22]. Building on this, the present study investigates the impact of incorporating 10% OC into the diet of Bísaro pigs, specifically examining its effects on the chemical composition and lipid profile of three fresh ham muscles: *biceps femoris* (BF), *semimembranosus* (SM), and *semitendinosus* (ST).

## 2. Materials and Methods

This study was conducted at the Experimental Swine Unit of the University of Trás-os-Montes and Alto Douro (UTAD, BioLab Sus) in Vila Real, Portugal. The handling of the animals adhered to Portuguese legislation on animal welfare in experimental research (Decree-Law No. 1/2019 of 10 January), the Directive 2010/63/EU of the European Parliament and the Council of 22 September 2010. The Animal Welfare Body (ORBEA) of the University of Trás-os-Montes and Alto Douro approved the study protocol under reference number 2253-e-DZ-2022.

### 2.1. Animals and Diets

The animals used in this study were the same as those described in Leite et al. [23]. Forty Bísaro pigs were used, starting with a live weight (LW) of 103.0 ± 3.476 kg. The animals were housed in pairs, with eight replicates per treatment for live weight and four replicates for feed intake. An initial acclimatization period of eight days was included to allow the animals to adapt to the facilities and diets. The experimental trial lasted for 12 weeks, during which the pigs were subjected to five dietary treatments: CD (Control Diet), COC (Control Diet + 10% Crude OC), TPOC (Control Diet + 10% Two-Phase OC), EOC (Control Diet + 10% Exhausted OC), and EOC-OO (Control Diet + 10% Exhausted OC + 1% Olive Oil).

The inclusion level of 10% for all OC types was determined considering the nutrient dilution effect in the base feed, the animal’s nutritional requirements, and their productive response. Given the extended duration of this trial, maintaining a consistent inclusion level ensured a balanced assessment of the impacts of the dietary treatments.

### 2.2. Slaughter Procedure

The 40 animals included in this study were randomly allocated to five dietary treatments. Due to the number of animals, it was not possible to slaughter them all at once. Therefore, they were divided into four slaughter batches, ensuring a balanced distribution of treatments across batches. The animals arrived in two shipments and were split into groups with different initial body weights. The first group, consisting of heavier animals, was assigned to slaughter batches 1 and 2, while the second group, composed of lighter animals, was allocated to batches 3 and 4. Once the pigs reached an average LW of 130–140 kg, they were slaughtered at the Bragança Municipal Slaughterhouse, following animal welfare standards and complying with EU Council Regulation (EC) No. 1099/2009 [24]. All animals underwent a 24 h fasting period before slaughter.

### 2.3. Chemical Composition and Physicochemical Analysis

After slaughter, the carcasses were divided and sent to the Carcass and Meat Quality Laboratory of ESA-IPB (Escola Superior Agrária, Instituto Politécnico de Bragança) to analyze meat composition and quality. Samples of the BF, SM, and ST muscles were taken for physical, chemical, and fatty acid analyses carried out in triplicate. The moisture content [25], ash [26], and protein [27] were determined following standard Portuguese procedures. The collagen content was measured based on the hydroxyproline concentration, established by the Portuguese Standard NP 1987/2002 [28]. The haem pigments were obtained using the reflectance technique of the exposed surface, using the Spectronic Unicam 20 Genesys spectrophotometer (SPEC-TRONIC 20 GENESYS, Thermo Fisher Scientific, Austin, TX, USA). The procedure is based on Hornsey’s approach [29], and the results are expressed in mg of myoglobin/g of fresh muscle.

### 2.4. Fatty Acid Analysis

The procedure for determining the lipid content was carried out at the Carcass and Meat Quality Laboratory of ESA-IPB, following the method previously described by Folch [30]. We used 25 g of meat to extract total lipids, while 50 g was used to determine the fatty acid profile. The transesterification of fatty acids was performed following the protocol established by Domínguez et al. [31]. In this process, 4 mL of sodium methoxide solution was added to the sample, followed by stirring for 5 min intervals for 15 min at room temperature. Subsequently, 5 mL of a solution of H_2_SO_4_ (50% in methanol) and 2 mL of distilled water were added, with continued stirring. The organic phase containing the fatty acid methyl esters was extracted using 2.35 mL hexane. The methyl esters were separated and quantified using a gas chromatograph (GC-Shimadzu 2010Plus; Shimadzu Corporation, Kyoto, Japan), which was equipped with a flame ionization detector, an AOC-20i automatic injector (Kyoto, Japan), and a Supelco SP™-2560 (Sant Louis, MO, USA) fused silica capillary column (100 m long, 0.25 mm internal diameter, 0.2 µm film thickness). The fatty acid contents were determined based on the areas of the chromatogram peaks and expressed as grams per 100 g of total fatty acid methyl esters. Additionally, the proportions of saturated fatty acids (ΣSFAs), monounsaturated fatty acids (ΣMUFAs), and polyunsaturated fatty acids (ΣPUFAs) were calculated, along with the n-6/n-3 and ΣPUFA ratios, as per the method outlined by Vieira et al. [32]. To evaluate lipid quality, the atherogenic index (IA) and thrombogenic index (IT) were calculated according to the methodology of Ulbricht and Southgate [33].

The selection of fatty acids in this study was based on their relevance to the lipid composition of pork and their consistent presence in the analyzed samples [34]. Only those reliably detected fatty acids that contributed significantly to the overall lipid profile were included in the statistical analysis and subsequent discussion.

### 2.5. Statistical Analysis

The data were tested for normality and homogeneity of variance using the Shapiro–Wilk test. We then examined the effects of treatment, muscle type, and their interaction on the chemical composition and fatty acid profiles. This was achieved using analysis of variance (ANOVA) with the general linear model (GLM) procedure, where these parameters were defined as dependent variables, and treatment and muscle type were considered fixed effects. The results are presented as mean values with the standard error of the mean (SEM). In cases where a significant effect (*p* < 0.05) was detected, the post hoc Tukey test was employed to compare the means.

To identify the key combinations (referred to as principal components) of the measured variables that explain the majority of the variability, we performed a principal component analysis (PCA). Each principal component was determined by the linear combination of the eigenvectors of the correlation matrix, with the eigenvalues indicating the amount of variance each component explains. All analyses were conducted using the statistical software JMP^®^ Pro 17.1.0, 2023 edition, from SAS Institute Inc.© (Cary, NC, USA).

## 3. Results and Discussion

### 3.1. Chemical Compositions of Diets

The nutritional characterization of each type of OC was described earlier by Paié-Ribeiro et al. [31,32]. The chemical compositions of the experimental diets showed significant variations among treatments, particularly in fiber content and lipid composition (Table 1).

The inclusion of OC resulted in a substantial increase in acid detergent fiber (ADF) and acid detergent lignin (ADL) compared to the CD. While the control diet had ADF (6.29%) and ADL (0.92%), the other treatments exhibited considerably higher values, ranging from 10.1 to 10.7% for ADF and 2.86 to 3.15% for ADL.

Regarding the fatty acid composition, notable differences were observed among the experimental diets. The MUFA content was significantly higher in the COC (42.4%) and TPOC (38.3%) diets compared to the control (28.0%), while the EOC (29.5%) and EOC-OO (36.1%) diets showed intermediate values.

Conversely, the PUFA contents were highest in the control diet (50.5%) and the EOC treatment (49.2%), followed by EOC-OO (43.3%). The lowest PUFA values were observed in the COC (37.6%) and TPOC (41.6%) diets. These results indicate that including COC and TPOC increased MUFA levels while reducing the PUFA content. Adding EOC and combining EOC-OO contributed to maintaining higher PUFA levels, like the control diet. The PUFA/SFA ratio was also reduced in the COC and TPOC treatments, indicating a possible modification in the lipid profile of animals fed these diets.

The inclusion of OC in pig diets presents both nutritional challenges and benefits. This agro-industrial by-product is characterized by a high fiber content, which can reduce nutrient digestibility. In this study, incorporating OC significantly increased ADF and ADL levels in experimental diets compared to the control, which could impact nutrient absorption and feed efficiency. Despite these challenges, OC also provides a valuable lipid source, which plays a crucial role in energy supply and modifying the fatty acid profile of pork [21,35].

The fatty acid composition of the diets had a direct impact on the lipid profile of the meat. Diets containing COC and TPOC increased MUFAs while reducing PUFAs. In contrast, EOC and its combination with olive oil (EOC-OO) helped maintain higher PUFA levels, similar to the control diet. These findings align with previous studies showing that dietary lipid sources influence fatty acid deposition in animal tissues, with implications for meat quality and consumer health [36].

The fatty acid profile of pork is a key factor in meat quality, affecting aspects such as texture, flavor, and nutritional value. Autochthonous pig breeds, such as Bísaro pigs, are known for their high intramuscular fat content, contributing to their distinctive sensory properties. Adjusting their diet can enhance the lipid composition of the meat without compromising its traditional characteristics [37]. Given the growing interest in improving pork’s nutritional quality, dietary strategies incorporating olive by-products offer a sustainable approach to modifying fat composition while promoting circular economy practices in the agri-food sector.

Despite the nutritional variability of OC, its inclusion at up to 10% in Bísaro pig diets did not compromise meat quality, highlighting its potential as an alternative feed ingredient. However, factors such as fiber composition, anti-nutritional compounds, and processing methods should be carefully considered to optimize its use in large-scale production [38,39,40,41]. Further research into the impact of OC on meat texture, oxidative stability, and consumer acceptance would provide a more comprehensive understanding of its role in pig nutrition.

### 3.2. Chemical Composition

The results of the analyses of the chemical composition of the BF, ST, and SM muscles shown in Table 2 reveal significant differences (*p* < 0.05) in the effects of the different experimental treatments, the muscles, and the interaction between the two on a_w_, protein, collagen, and haem pigments. The results were variable between the muscle groups studied, and it is essential to emphasize that each muscle plays a role in its composition, and even different sections of the same muscle can vary in terms of chemical composition.

Water activity (a_w_) was significantly affected by diet (*p* = 0.045) and muscle (*p* < 0.001), with a significant interaction between the two (*p* = 0.001). Concerning humidity, there were significant effects on this parameter between the different diets and muscle groups studied. The diets significantly affected this parameter, with the POC group having the highest moisture content (72.11), followed by the EOC, COC, EOC-OO, and CD groups (71.05, 69.66, 68.58, and 67.42, respectively). Moisture content among the muscle groups was highest in the BF group (71.83), followed by SM and ST (70.52 and 71.83, respectively).

The ash content (%) was significantly higher (*p* = 0.023) in the groups that received OC incorporation (mean 1.17 ± 0.048) compared to the CD group (1.02). This parameter also revealed significant differences between the muscle groups, being higher in the BF muscle (1.62), followed by the ST muscle (1.18) and SM (0.62).

The fat content (%) revealed significant results. Surprisingly, the groups that received the addition of OC had lower muscle fat contents (average of 6.47% ± 0.553) compared to the CD group (10.37), with the ST muscle having the highest fat content (11.15), followed by the SM (6.75) and BF (3.85) muscles. Notably, the muscle protein content was also higher in the groups that received the addition of OC, showing a higher average of 21.01% compared to the CD group (19.37). As for the muscle group, BF had the highest protein content (22.27), followed by SM (20.17) and ST (19.61).

The diet and muscle group also significantly affected the hydroxyproline content (mg/g) and consequently collagen (%) (*p* < 0.05). The CD and COC groups had the highest values of this amino acid (0.14 ± 0.006) and collagen (average of 1.13 ± 0.049), followed by the EOC-OO, TPOC, and EOC groups. Concerning the muscle group and following the same logic, the ST muscle had the highest hydroxyproline and collagen content, followed by the BF and SM.

Haem pigments (mg/g) were also significantly affected by diet (*p* < 0.001) and muscle group (*p* < 0.001). The COC group had the highest values (2.55), followed by the CD, EOC, TPOC, and EOC-OO groups (2.31, 2.07, 2.00, and 2.00, respectively). The haem pigment content was highest in the SM muscle, followed by the BF (2.01) and ST (1.89).

Meat quality encompasses aspects related to both consumer acceptance and technological characteristics. Muscle fiber characteristics are one of the main factors influencing meat quality, as they affect the meat’s color, water retention capacity, pH, and tenderness [42,43,44]. As listed in Table 1, there is a clear difference between the treatments, muscle types, and their interaction (*p* < 0.05) in the physico-chemical parameters analyzed. The values for a_w_, protein, collagen, and haem pigments significantly differed in the interaction between the treatments and the three muscles studied (BF, ST, and SM). The lack of statistical significance of the interaction between treatments and muscles in the other parameters suggests that the effects of the different treatments do not vary significantly between the different muscles. In other words, the treatment response is consistent between the muscles studied.

Table 3 shows differences in how the diet affects the chemical composition of each muscle group analyzed. The chemical composition of the BF muscle displays distinct profiles for each measured parameter. No significant differences were observed in a_w_ (*p* = 0.157). As for protein, this muscle group shows essential results. Notably, the groups that received the addition of OC had the highest levels of this parameter, with a higher average of 22.61 ± 0.277 compared to the CD group (20.91). The collagen content showed a trend towards significance (*p* = 0.057), with the COC group showing the highest value, followed by the CD, EOC-OO, EOC, and TPOC groups (1.19, 0.71, 0.66, and 0.55, respectively). The different diets also significantly affected (*p* = 0.041) the composition of haem pigments in this muscle group, with the COC group showing the highest value, followed by the TPOC, CD, EOC-OO, and EOC groups (2.33, 2.01, 1.99, 1.96, and 1.75 mg/g, respectively).

The chemical composition of the ST muscle showed some different results to the BF muscle. Concerning a_w_, significant differences were observed (*p* = 0.023) in the animals that received the EOC-OO treatment, which showed the highest content of this parameter (0.96), followed by COC (0.95) and the other treatments (0.94). Although the total fat content showed a trend towards significance (*p* = 0.071), the highest values were recorded in the animals that received the CD (17.48), while the TPOC treatments showed the lowest values (8.35). Similarly, protein showed a trend towards significance (*p* = 0.074), with the animals in the CD group showing the lowest levels (17.41). In comparison, the OC treatments showed a higher average (20.16), which aligns with the results obtained for the BF muscle. The hydroxyproline content also showed significant results (*p* = 0.016), with the animals in the CD group recording the highest values (0.20), followed by EOC-OO (0.19), TPOC (0.18), COC (0.16), and EOC (0.14). Similarly, the collagen content followed a significant pattern (*p* = 0.015), with the highest values being observed in the CD group (1.59), followed by EOC-OO (1.54), TPOC (1.41), COC (1.31), and EOC (1.14). The haem pigments also showed significant results (*p* < 0.001), with the animals in the COC group showing the highest muscle pigmentation (2.77 mg/g), followed by the CD (2.08), EOC (1.89), TPOC (1.63), and EOC-OO (1.07) groups.

The chemical analyses obtained from the SM muscle reveal notable variations between the dietary groups considered. The a_w_ showed significant differences (*p* < 0.05), with the CD group recording the highest values (0.97). In contrast, the EOC and EOC-OO groups obtained slightly lower values (0.96), and the TPOC group recorded the lowest (0.95). As for the protein content, the EOC and TPOC groups stood out with the highest values (21.37 and 20.89, respectively), while the EC, EOC-OO, and COC groups maintained an average of 19.53 ± 0.373. Regarding haem pigments (mg/g), the EOC-OO group had the highest value (2.97 mg/g), while the TPOC group had the lowest value (2.36 mg/g).

#### Principal Component Analysis of Chemical Composition

Principal component analysis (PCA) enabled identifying and interpreting relationships between the BF, SM, and ST muscles, considering the analyzed variables: aw, moisture, ash, total fat, protein, collagen, and haem pigments. This approach facilitated a clearer and more objective understanding of the observed interactions and patterns. The first two principal components (PC1 and PC2) jointly explained 69.3% of the total variance in the data, with 43.1% attributed to PC1 and 26.2% to PC2, indicating that these axes capture most of the variation observed between the muscle groups.

**Figure 1:**
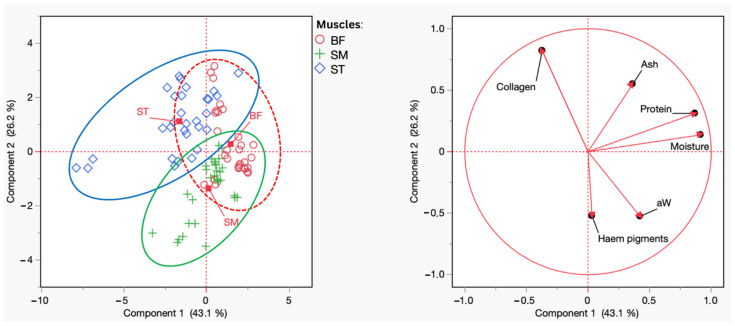
Principal component analysis (PCA) scores and loading plots of a_w_, moisture, ash, total fat, protein, collagen, and haem pigments of BF (red), SM (green), and ST (blue).

Figure 1 presents the PCA results in the form of a biplot. The ST muscle stands out on the left side of the plot, associated with variables related to connective tissue, such as collagen. In contrast, the BF is positioned centrally, suggesting it shares characteristics with both the SM and the ST. The SM occupies an intermediate position, reflecting chemical characteristics that combine aspects of the other two muscles, with a notable emphasis on higher protein and ash contents.

These results suggest significant differences in the chemical compositions of the studied muscles, highlighting that, although these three muscles are anatomically close and have related functions, they exhibit distinct chemical compositions. The ST, with its higher collagen content, possesses characteristics associated with a greater proportion of connective tissue, which may influence its texture and technological functionality. On the other hand, the centrally positioned BF exhibits a composition that reflects intermediate characteristics between the SM and ST. At the same time, the SM presents a balanced chemical profile between the two extremes.

The observed separation between the SM and ST, with the BF occupying a central position, reflects the influence of the intrinsic characteristics of each muscle and reinforces the importance of considering these differences when selecting these cuts for specific purposes, whether in industrial processing or direct consumption.

A product is classified as fresh if it has an a_w_ of 0.90 or above. Fresh meat typically has an a_w_ of around 0.98, which decreases during processing. For example, the a_w_ of Bísaro ham has been reported to reduce to an average of 0.85, with values ranging from 0.72 to 0.92 [45,46]. Similar a_w_ values have been documented in different cuts of Bísaro pigs, such as the loin (0.98), shoulder (0.97), and leg (0.97) [47]. In our study, the a_w_ values were consistent with these findings, with an average of 0.95 ± 0.003 in the ST muscle and 0.96 ± 0.002 in the SM muscle. Both muscles are, therefore, classified as fresh products with a high water activity (a_w_ > 0.90). The high moisture content and elevated aw levels suggest that the meat maintains its juiciness and tenderness, characteristics that are essential for consumer acceptability and overall eating quality.

The protein contents in the muscle groups receiving OC supplementation were notably higher in the BF (22.61) and ST (20.16) muscles compared to the control group (average 19.61). These results align with previous studies; Park et al. [48] reported similar protein levels (22.88) of the *Longissimus dorsi* (LM) muscle of pigs fed diets supplemented with 3% olive oil into the diet. Likewise, Leite et al. [49] found a similar trend in dry-cured Bísaro loin incorporating different OC types. Kameník et al. [50] also observed comparable protein contents in the BF (21.54), ST (20.64), and SM (22.39) muscles of selected pigs. However, unlike the BF and ST muscles, the protein content in the SM muscle did not follow the same increasing trend. These variations are likely due to anatomical differences, as these three muscles belong to the posterior thigh muscle group but differ in function and positioning [50]. The BF is an internal muscle, while the ST and SM are external, making them subject to different metabolic demands. Additionally, variations in protein deposition within different muscle sections may also contribute to these differences [49].

The intramuscular collagen content is a crucial factor in meat texture, as it influences firmness and tenderness [36]. In our study, the ST muscle exhibited significantly higher collagen levels compared to the BF and SM muscles. The values observed (average of 1.40 ± 0.092) were considerably higher than those reported by Kameník et al. [50] (0.47 ± 0.15). An increased collagen content can affect meat tenderness, as higher collagen levels often correlate with increased toughness. However, collagen also plays a functional role in muscle structure and can contribute to the perceived mouthfeel of cooked meat.

Meat color is a key attribute influencing consumer preference and is primarily determined by the haem pigment concentration, which varies across breeds and muscle types [51]. In our study, significant differences in haem pigment content were observed among the three muscles, with the SM muscle exhibiting the highest levels, followed by the BF and ST muscles. The differences in pigment content between the SM muscle and the other two muscles were 0.77% and 0.65%, respectively. Since haem pigments contribute to the red coloration of meat, their concentration directly impacts consumer perceptions of freshness and quality. Additionally, higher haem pigment levels may be associated with greater iron bioavailability, which is nutritionally relevant, as haem iron is more efficiently absorbed than non-haem iron from plant sources. Therefore, changes in pigment content not only affect meat appearance but also have potential health implications, particularly for populations at risk of iron deficiency.

From a health standpoint, the chemical alterations noted in this study carry significant implications for both meat quality and human nutrition. The rise in protein content in specific muscle groups indicates a possible improvement in the meat’s nutritional profile, especially in supplying essential amino acids vital for muscle repair, growth, and general well-being. This is consistent with research underscoring meat as a key source of high-quality protein and important nutrients [52].

Variations in the fatty acid composition indicate that dietary interventions can effectively modulate the lipid profile of pork. Adjusting the balance between saturated FA, MUFA, and PUFA can lead to health benefits, such as improved cardiovascular health. Studies have demonstrated that dietary manipulation, including the incorporation of specific lipid sources, can alter the fatty acid profile of pork, potentially providing consumers with healthier meat options [53].

Furthermore, the observed differences in haem pigment content may impact iron bioavailability, potentially enhancing the nutritional quality of the meat. Meat contains haem iron, which is more efficiently absorbed by the body compared to non-haem iron present in plant-based foods. This makes meat a particularly valuable source of iron, especially for individuals at risk of iron deficiency [54].

These findings underscore the importance of dietary strategies in modulating the meat composition, with potential benefits not only for product quality but also for human health. By tailoring animal diets, it is possible to produce meat products that align more closely with nutritional guidelines and consumer health interests.

### 3.3. Fatty Acid Profile

The results of the analyses of the fatty acid composition of the BF, ST, and SM muscles shown in Table 4 reveal significant differences between the different experimental treatments in the muscles and the interaction between them. In both cases, palmitic acid (C16:0) was the most abundant SFA, oleic acid (C18:1n-9) the most abundant MUFA, and linoleic acid (C18:2n-6) the most abundant PUFA.

C10:0, C15:0, C16:0, and C18:0 were the SFAs that showed significant differences (*p* < 0.05). Concerning the C10:0 acid content, the COC and TPOC groups had the highest acid contents, followed by the CD (0.05), EOC (0.04), and EOC-OO (0.04) treatments. C15:0 acid showed significant effects on the different diets (*p* = 0.024), muscle (*p* < 0.001), and the interaction between the two (*p* = 0.008). The group receiving the TPOC diet had the highest content of this acid (0.20), followed by the COC (0.13), EOC (0.08), EOC-OO (0.05), and CD (0.04) groups. The BF muscle had the highest content of this acid (0.23), followed by the SM (0.04) and ST (0.02) muscles. The C16:0 acid was the most abundant within the SFAs, accounting for 66.08% of the total SFAs. It was not affected by the different treatments (*p* = 0.078), with an average of 24.50 between the groups; however, in the ST and SM muscles (average of 24.65 ± 0.080), the content of this acid was significantly higher compared to the BF muscle (24.12).

The content of stearic acid (C18:0) was significantly higher (*p* = 0.008) in the groups that received the EOC treatment (11.38). Among the muscle groups, it was more abundant in the ST muscle (11.51). Including OC in the animals’ diet did not influence the total SFA content. However, significant differences (*p* < 0.001) were observed for SFA between the muscle groups, with the ST muscle being the most abundant in SFA (37.91).

MUFA was the most prevalent fatty acid group in all the treatments and muscles analyzed. Palmitoleic acid (C16:1n-7) was significantly affected by the diets and muscles, being more abundant in the animals given the COC diet (3.58) and lower in the other groups (mean 3.14 ± 0.057). The content of this acid varied significantly between the three muscles analyzed, with BF having the highest content (3.51), followed by SM (3.24) and ST (2.94). Margaroleic acid (C17:1n-7) was significantly affected by the different diets (*p* = 0.014), being more abundant in the animals that received the CD treatment (0.24), with no significant differences between the muscle groups studied. The content of elaidic acid (9t-C18:1) varied between 0.14% and 0.17% between the different diets (*p* = 0.486) and 0.12% (ST) and 0.17% (BF and SM) (*p* = 0.004) between the muscles. The main fatty acid (C18:1n-9) showed significant differences between treatments and the interaction between treatments and muscles (*p* < 0.001), with values ranging from 51.11 to 49.04% for the different treatments and 50.48–50.08% for the three muscles. Eicosenoic acid (C20:1n-9) was significantly affected by the diets (*p* = 0.004), ranging from 0.69% (TPOC) to 0.83% (CD). The total MUFA content was significantly affected by the different diets, muscle groups, and their interaction, ranging from 53.33 to 55.69% for the diets and 54.45 to 55.25% for the muscles.

PUFA ranged from 7.31 to 0.77% and 7.64 to 8.59% in the diets and muscles, respectively. The TPOC group had a higher total PUFA value (9.77). Linoleic acid (C18:2n-6) and alpha-linolenic acid (C18:3n-3) were also significantly higher (*p* < 0.001) in this same group (8.18 and 0.29, respectively). As for the muscle group, linoleic acid was not found at significant levels among the three muscles studied (*p* = 0.127). However, the ST and SM muscles had significantly (*p* < 0.001) higher levels of alpha-linolenic acid (mean 0.27 ± 0.005) compared to the BF (0.02). The content of eicosadienoic acid (C20:2n-6) varied significantly (*p* = 0.001) between 0.24 and 0.28% in the three muscles analyzed. Arachidonic acid (C20:4n-6) showed significant values in diets and muscles and their interaction. Once again, the TPOC group had the highest levels of this acid (0.76). As for the muscle group, BF had the highest content of this acid (0.82), followed by SM (0.62) and ST (0.10).

The PUFA/SFA and n-6/n-3 PUFA ratios were calculated to assess the fat’s quality. Concerning PUFA/SFA, the values varied between 0.20 and 0.27% and between 0.20 and 0.23% for the diets and muscles evaluated, respectively, with significant differences obtained between the different treatments, muscles, and their interaction (*p* < 0.05).

In our study, the n-6/n-3 PUFA ratio values varied from 20.50 to 21.88 between the treatments and 20.77 to 21.44 between the muscles analyzed, but without statistical significance (*p* > 0.05).

The IA and the IT, developed by Ulbritcht and Southgate in 1991, are associated, respectively, with the potential for atherogenicity and thrombogenicity [33]. The IA indicates the relationship between the sum of SFAs and the sum of unsaturated fatty acids (UFAs). Among the SFAs, the C12:0, C14:0, and C16:0 classes (except C18:0) are pro-atherogenic, as they favor the adhesion of lipids to the cells of the circulatory and immune systems. UFAs have an anti-atherogenic effect, helping to reduce plaque accumulation and levels of phospholipids, cholesterol, and esterified fatty acids. Thus, foods with lower IA can contribute to reducing total cholesterol and LDL-C levels in human blood [55].

Based on the results, the IA and IT showed no significant differences between the treatments, with averages of 0.46 ± 0.461 for the IA (*p* = 0.344) and values of 1.11–1.15 for the IT (*p* = 0.199). However, when analyzed for the different muscles, there was statistical significance (*p* < 0.001 for IT and *p* = 0.001 for IA), with the ST muscle showing the highest values for both indices (IA = 0.47 and IT = 1.17). These results are within the usual range for meat (0.165 and 1.32 for IA and between 0.288 and 1.694 for IT) [55]. A low AI value is desirable as it indicates a lower proportion of SFA and reduces plaque formation in the arteries, which is beneficial for cardiovascular health [55,56]. The same was observed for the IT index, with values between 1.11 and 1.15 and between 1.09 and 1.17 found for the treatments and muscles, respectively. There were no statistical differences between the different treatments (*p* = 0.199). Still, there was a significant difference (*p* < 0.001) in this index between the other muscles, with the ST muscle also obtaining the highest value (1.17).

There were no significant differences (*p* > 0.05) between the different treatments in the h/H ratio (hypocholesterolemic/hypercholesterolemic index) in this study. Still, the values significantly differed between the muscle groups (*p* = 0.007), with the BF muscle having the highest content (2.31), followed by the SM (2.27) and ST (2.23).

Certain fatty acids, such as C14:1n-5, C16:1n-9, C18:1n-7, C18:4n-3, C20:0, C20:3n-5, and C20:5n-3, were not included in this study as their concentrations were below the detection limit in several samples. Given these limitations, only the fatty acids that were consistently detectable and present at sufficient concentrations were considered and are presented in the table below.

Various dietary elements can impact the fatty acid composition in pig tissues, influencing several health-related indices. In research conducted by Sońta et al. [57], substituting part of the genetically modified soybean meal with pea seeds and rapeseed meal in complete feed mixtures for growing and finishing pigs led to a diet-induced decrease in the atherogenic index (IA), thrombogenic index (IT), and saturation (S/P) values in the pork from the experimental group compared to the CD. These findings suggest that dietary adjustments, such as replacing certain feed components, can have a beneficial impact on the fat profile and cardiovascular health indices in pigs. Overall, these indices are primarily influenced by the balance of SFA, MUFA, and PUFA in the diet. Pork generally exhibits higher IA and IT values when compared to poultry, indicating a less favorable fatty acid profile for cardiovascular health [58]. This highlights the importance of carefully modulating the fat composition in pig diets to improve meat quality and its potential health benefits for consumers.

Table 5 shows the significant effects (*p* < 0.05) of the different treatments on the composition of C15:0, C18:1n-9, C18:2n-6, C20:4n-6, MUFAs, PUFAs, and PUFA/SFAs in the three muscles studied. The BF muscle showed significant effects (*p* = 0.046) on the pentadecanoic acid (C15:0) content, with the TPOC treatment showing the highest value (0.53) compared to CD (0.06). Oleic acid (C18:1n-9), the primary MUFA, also varied significantly (*p* < 0.0001) in the BF muscle, with the highest content found in the group receiving the CD treatment (52.72). Concerning arachidonic acid (C20:4n-6), significant effects (*p* < 0.05) were found in the BF and ST muscles, with values ranging from 0.43 to 1.74% and 0 to 0.26%, respectively. The total MUFA content was also significantly different (*p* < 0.001) between treatments in the BF muscles, with the highest value in the CD group (57.36). The SM muscle showed a trend towards significance (*p* = 0.076), with the highest value in the TPOC group (55.00). The total PUFA content showed significant variations (*p* < 0.05) in the BF (6.48–12.21%) and ST (6.84–8.34%) muscles, registering the highest levels in the sets that incorporated OC compared to the CD group. The PUFA/SFA ratio also reflected this trend in the BF and ST muscles, revealing significant effects (*p* < 0.05).

In summary, BF was the muscle that exhibited the most significant changes in fatty acid profile, demonstrating greater sensitivity to dietary variations, particularly in diets containing TPOC. These findings suggest that the lipid composition of BF may be more easily modulated through dietary interventions, making it a promising target for future studies aimed at enhancing meat nutritional quality.

Using by-products in pig diets has been extensively studied to enhance the nutritional quality of meat while adding value to agro-industrial waste. Among these by-products is OC—commonly produced in the Trás-os-Montes region—with a high content of MUFA, mainly oleic acid, making it a promising ingredient for inclusion in pig diets [59]. Research indicates that incorporating this by-product can alter the lipid profile of the animals’ fat and muscle tissues, thereby improving meat [60,61].

Although the MUFA profile showed significant variation in the BF muscle (TPOC: 51.52; CD: 57.36), its content fell within the range documented for several local breeds, including the Preto Alentejano [16] and Iberian pigs [62,63]. This MUFA proportion appears to be a common trend among native pig breeds from Southern Europe. However, lower MUFA values have been observed in Alentejano pigs [64].

Notable variations in PUFA content were identified in the BF and SM muscles. In the BF muscle, PUFA levels fluctuated between 6.48% in the CD treatment and 12.21% in the TPOC treatment. A comparable pattern was observed in the SM muscle, with the PUFA content ranging from 6.84% in the CD treatment to 8.09% in the TPOC treatment. PUFA levels are influenced by multiple factors, including dietary fat composition and structure, fatty acid synthesis, conversion rates into other fatty acids and metabolites, and the proportion oxidized for energy consumption [11]. The inclusion of OC, which is rich in MUFA, in animal diets has been reported to reduce the PUFA content in red blood cell membranes [11,65]. The PUFA values recorded for Bísaro pigs in this study fall within the range reported in the literature (refs). However, higher PUFA levels have been observed in Pietrain pigs fed OC-supplemented diets (mean 14.24%) [11], as well as in Iberian pigs (15.51% in subcutaneous dorsal fat) [61] and Alentejano pigs (15.9%) [64].

The PUFA/SFA ratio differed significantly between the BF and ST muscles. However, compared to the recommended value of 0.4 set by the Department of Health and Social Care [66], the values observed in this study (ranging from 0.18 to 0.25) were below the suggested threshold. The PUFA/SFA ratio recorded for Bísaro pigs’ dorsal fat was more favorable than those reported in other pig breeds not supplemented with OC [63,64,67,68]. The dietary intake of n-3 and n-6 PUFA plays a crucial role in meat quality, particularly in lean pig breeds, and directly affects human health [68]. A lower n-6/n-3 PUFA ratio has been associated with multiple health benefits, including better cardiovascular health and reduced inflammatory responses.

As monogastric animals, pigs have a strong ability to reflect the composition of dietary fats in their tissues. This means that the inclusion of specific fat sources in the diet, such as OC, can directly influence the lipid characteristics of the meat, including the proportion of fatty acids and its organoleptic properties [48]. For instance, it has been observed that diets enriched with various lipid sources, including conjugated linoleic acid (CLA), can increase the concentration of this compound in pig tissues, offering potential benefits to human health, such as anti-cancer, anti-diabetic, and anti-atherogenic effects [69,70].

Despite these advantages, the results regarding the inclusion of OC in pig diets are not always consistent. Fatty acid analysis in BF, ST, and SM muscles revealed that, although OC is rich in oleic acid, the content of this fatty acid in the tissues does not always increase in direct proportion to its inclusion in the diet. In fact, in some instances, the control group had higher levels of oleic acid in the BF muscle than the groups fed diets supplemented with OC. This discrepancy can be attributed to various metabolic and nutritional factors. For example, fatty acid metabolism in pigs is influenced by the overall composition of the diet and the interaction between nutrients. The processing and digestibility of OC can also affect the bioavailability of its bioactive compounds, reducing its effectiveness as a source of oleic acid when compared to more balanced and easily digestible control diets. Additionally, compounds found in OC can interfere with the absorption and deposition of nutrients in tissues [71].

Previous studies have indicated that including up to 10% OC in pig diets does not significantly affect parameters such as pH or meat color. However, it may lead to a reduction in intramuscular fat content. Furthermore, the use of olive by-products in feed has been associated with an increased profile of unsaturated fatty acids without compromising sensory acceptance or consumer perception [11].

On the other hand, supplementation with other compounds, such as conjugated linoleic acid (CLA), has shown more consistent results. For example, diets containing 2% CLA-enriched oil resulted in 14.9 mg of CLA/g of fatty acids in the adipose tissue of Large White pigs, while control diets showed undetectable levels of CLA [72]. Similar results were observed in pigs fed 1% CLA, which showed 5.5 mg of CLA/100 g of fatty acids in the muscle [73].

In general, the lipid composition of pig tissues can be manipulated through the diet, offering opportunities for the pig industry to provide meat products with enhanced nutritional value and health benefits for consumers [74]. However, to maximize the utilization of by-products like OC, it is essential to gain a deeper understanding of the factors affecting the bioavailability and metabolism of nutrients in pigs. This knowledge is key to achieving consistent enhancements in the nutritional quality of the meat.

#### Principal Component Analysis of the Fatty Acid Profile

**Figure 2:**
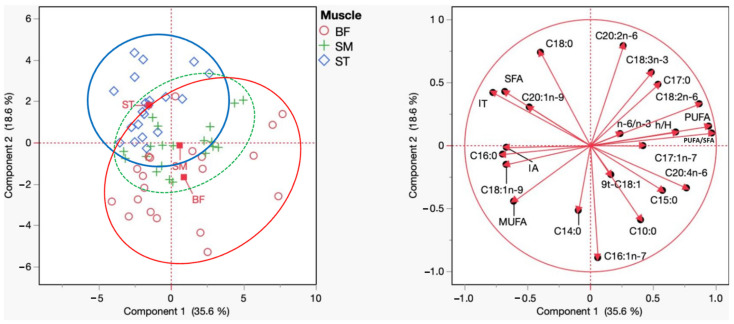
Principal component analysis (PCA) scores and loading plots of the fatty acid profiles of BF (red), SM (green), and ST (blue). SFA—Saturated Fatty Acid; MUFA—Monounsaturated Fatty Acid; PUFA—Polyunsaturated Fatty Acid; n-6/n-3 (∑ omega-6) (∑ omega-3). IA—Index of Atherogenicity; IT—Index of Thrombogenicity; h/H—Hypocholesterolemic/Hypercholesterolemic Index.

Figure 2 presents that the principal component analysis (PCA) performed on the fatty acid profile of the BF, SM, and ST muscles revealed significant differences in lipid composition among these muscle groups. The first principal component (Component 1) explained 35.6% of the variance, while the second principal component (Component 2) accounted for 18.6%, totaling 54.2% of the variance explained by the first two components. This distribution suggests the data have a clear structure, allowing for differentiation between the muscles based on their fatty acid profiles.

The BF muscle stood out in the PCA, positioning itself distinctly from the SM and ST muscles, indicating a significantly different fatty acid composition. This separation can be attributed to its higher concentration of MUFA, such as C18:1n-9 (oleic acid), and the presence of PUFA, such as C18:2n-6 (linoleic acid). BF also exhibited a higher PUFA/SFA ratio than the other muscles, which may reflect a greater influence of the diets provided, particularly those enriched with OC and olive oil.

Conversely, the SM and ST muscles were positioned closer together in the PCA space, suggesting that they share similar characteristics in terms of fatty acid composition. Both muscles exhibited a higher contribution of SFA, such as C16:0 (palmitic acid) and C18:0 (stearic acid), and a lower presence of MUFA and PUFA compared to BF. This similarity may be related to their function and anatomical location, which could subject them to similar lipid metabolism processes.

Additionally, the PCA highlighted the importance of specific fatty acids, such as C18:1n-9 and C18:2n-6, which significantly contributed to the differentiation of muscle groups. These fatty acids are well-known for their beneficial health effects, such as reducing LDL cholesterol and improving cardiovascular health, further emphasizing the relevance of evaluating muscle lipid profiles in nutritional studies.

In summary, the PCA demonstrated that the BF muscle possesses a distinct fatty acid profile, with a greater influence of MUFA and PUFA, while the SM and ST muscles share a more similar composition, with a higher predominance of SFA. These results highlight the importance of considering differences between muscle groups when assessing the impact of diets on meat lipid profiles, with BF emerging as a particularly responsive muscle to dietary interventions.

The nutritional composition of pork can be significantly modulated through dietary interventions, influencing its protein content, fatty acid profile, and iron bioavailability. These modifications not only enhance the meat’s nutritional value but may also provide health benefits to consumers [75,76,77].

Studies have shown that supplementing pig diets with n-3 PUFA, such as those found in flaxseed, significantly increases the levels of alpha-linolenic acid and eicosapentaenoic acid in pork, thereby improving its nutritional quality [78]. Similarly, the inclusion of *Chlorella vulgaris* in pig feed has been found to increase the n-3 PUFA content while reducing the n-6:n-3 ratio, further enhancing the meat’s lipid profile [79].

These findings highlight that dietary adjustments in pig nutrition can serve as an effective strategy to optimize pork quality, making it a more balanced and nutritious protein source. Furthermore, such strategies may have positive implications for human health, particularly in diets aimed at improving essential fatty acid intake and reducing the risk of cardiovascular diseases.

## 4. Conclusions

The results of this study indicate that incorporating up to 10% of different types of OC into pig feed leads to variations in specific physicochemical properties of the muscles studied, while others remain unaffected. Significant differences were observed in parameters such as moisture content, protein, collagen, and haem pigments, whereas ash, total fat, and SFA remained unchanged. TPOC stood out for having the highest moisture and PUFA contents and the highest PUFA/SFA ratio, suggesting its potential to improve the lipid profile of meat. Conversely, EOC showed a higher content of haem pigments in two muscles, which may positively influence the color and antioxidant potential of the meat.

Overall, the findings suggest that using OC in pig diets is a viable strategy from both economic and environmental perspectives, as it promotes the valorization of by-products from the olive oil industry without significantly compromising the quality of the meat produced. This study underscores the importance of sustainable practices in livestock farming and opens avenues for future research into the impacts of different agro-industrial by-products on meat quality.

## Figures and Tables

**Table 1:** Ingredient compositions of the experimental diets and fatty acid compositions.

			Diets		
	CD	COC	TPOC	EOC	EOC-OO
Ingredients (g/100 g, as fed basis)					
Olive cake	0	10	10	10	10
Olive oil	0	0	0	0	1
Chemical composition (% dry matter)					
Dry matter	98.0	98.3	98.1	98.2	98.3
Organic matter	94.0	94.2	93.6	94.2	94.1
Neutral detergent fiber	17.8	23.3	23.3	23.9	23.1
Acid detergent fiber	6.29	10.7	10.4	10.4	10.1
Acid detergent lignin	0.92	3.15	2.86	3.07	2.95
Crude protein	15.4	13.9	13.5	14.3	14.0
Crude fat	4.63	5.19	4.82	3.99	4.88
Fatty acids (g/100 g)					
ΣSFA	21.5	20.0	20.1	21.3	20.6
ΣMUFA	28.0	42.4	38.3	29.5	36.1
ΣPUFA	50.5	37.6	41.6	49.2	43.3
PUFA/SFA	2.34	1.88	2.07	2.31	2.11
n-6/n-3	17.6	16.7	16.7	17.1	16.9

CD—Control Diet; COC—Control Diet + 10% Crude Olive Cake; TPOC—Control Diet + 10% Two-Phase Olive Cake; EOC—Control Diet + 10% Exhausted Olive Cake; EOC-OO—Control Diet + 10% Exhausted Olive Cake + 1% Olive Oil; SFA—Saturated Fatty Acid; MUFA—Monounsaturated Fatty Acid; PUFA—Polyunsaturated Fatty Acid; n-6/n-3 (∑ omega-6) (∑ omega-3).

**Table 2:** Chemical compositions of BF, ST, and SM muscles from the Bísaro pig breed. Effect of diets with olive cake, muscles, and interaction between diets and muscles.

	Diets					Muscles			SEM	*p*-Value		
	CD	COC	TPOC	EOC	EOC-OO	BF	ST	SM		Diets	Muscles	Diets × Muscles
a_w_	0.96	0.96	0.96	0.95	0.96	0.96	0.95	0.96	0.001	0.045	<0.001	0.001
Moisture (%)	67.42 ^c^	69.66 ^abc^	72.11 ^a^	71.05 ^ab^	68.58 ^bc^	71.83 ^a^	66.95 ^b^	70.52 ^a^	0.497	<0.001	0.008	0.804
Ash (%)	1.02 ^b^	1.17 ^a^	1.16 ^a^	1.23 ^a^	1.11 ^ab^	1.62 ^a^	1.18 ^b^	0.62 ^c^	0.048	0.023	<0.001	0.979
Total fat (%)	10.37 ^a^	6.90 ^b^	5.42 ^b^	5.73 ^b^	7.82 ^ab^	3.85 ^c^	11.15 ^a^	6.75 ^b^	0.553	0.002	<0.001	0.138
Protein (%)	19.37	20.75	21.31	21.14	20.84	22.27	19.61	20.17	0.199	0.0002	<0.001	0.035
Hydroxyproline (mg/g)	0.14 ^a^	0.14 ^ab^	0.11 ^bc^	0.10 ^c^	0.12 ^abc^	0.10 ^b^	0.17 ^a^	0.094 ^b^	0.006	0.008	<0.001	0.183
Collagen (%)	1.15	1.11	0.89	0.78	0.98	0.8	1.4	0.75	0.049	<0.001	<0.001	<0.001
Haem pigments (mg/g)	2.31	2.55	2.00	2.07	2.00	2.01	1.89	2.66	0.059	<0.001	<0.001	<0.001

a_w_—Water Activity. Haem pigments in mg myoglobin/g fresh muscle. CD—Control Diet; COC—Control Diet + 10% Crude Olive Cake; TPOC—Control Diet + 10% Two-Phase Olive Cake; EOC—Control Diet + 10% Exhausted Olive Cake; EOC-OO—Control Diet + 10% Exhausted Olive Cake + 1% Olive Oil; BF—*Biceps femoris*; ST—*Semitendinosus*; SM—*Semimembranosus*; SEM—Standard Error of the Mean. Different lowercase letters (a, b, c) in the same row correspond to significant differences between diets (*p* < 0.05). ANOVA followed by a post hoc Tukey test.

**Table 3:** Chemical compositions of BF, ST, and SM muscles from the Bísaro pig breed. Effects of diets with olive cake.

	Muscles	Diets					SEM	*p*-Value
		CD	COC	TPOC	EOC	EOC-OO		
a_w_	BF	0.96	0.96	0.97	0.95	0.96	0.005	0.157
	ST	0.94 ^c^	0.95 ^ab^	0.94 ^bc^	0.94 ^c^	0.96 ^a^	0.003	0.023
	SM	0.97 ^a^	0.97 ^a^	0.95 ^b^	0.96 ^b^	0.96 ^b^	0.002	<0.001
Protein (%)	BF	20.91 ^b^	22.69 ^a^	22.58 ^a^	22.21 ^a^	22.95 ^a^	0.277	<0.001
	ST	17.41 ^b^	20.21 ^a^	20.47 ^a^	19.85 ^a^	20.12 ^a^	0.800	0.074
	SM	19.80 ^b^	19.36 ^b^	20.89 ^a^	21.37 ^a^	19.44 ^b^	0.373	0.002
Collagen (%)	BF	0.86	1.19	0.55	0.66	0.71	0.153	0.057
	ST	1.59 ^a^	1.31 ^bc^	1.41 ^abc^	1.14 ^c^	1.54 ^ab^	0.092	0.015
	SM	1.02	0.82	0.72	0.54	0.68	0.155	0.293
Haem pigments (mg/g)	BF	1.99 ^ab^	2.33 ^a^	2.01 ^ab^	1.75 ^b^	1.96 ^b^	0.123	0.041
	ST	2.08 ^b^	2.77 ^a^	1.63 ^c^	1.89 ^bc^	1.07 ^d^	0.103	<0.001
	SM	2.86 ^ab^	2.56 ^bc^	2.36 ^c^	2.57 ^bc^	2.97 ^a^	0.119	0.008

a_w_—Water Activity. Haem pigments in mg myoglobin/g fresh muscle. CD—Control Diet; COC—Control Diet + 10% Crude Olive Cake; TPOC—Control Diet + 10% Two-Phase Olive Cake; EOC—Control Diet + 10% Exhausted Olive Cake; EOC-OO—Control Diet + 10% Exhausted Olive Cake + 1% Olive Oil; BF—*Biceps Femoris*; ST—*Semitendinosus*; SM—*Semimembranosus*; SEM: Standard Error of the Mean. Different lowercase letters (a, b, c) in the row column correspond to significant differences between diets (*p* < 0.05). ANOVA followed by a post hoc Tukey test. Only variables with statistically significant interactions are presented.

**Table 4:** Fatty acid profiles (g/100 g) of BF, ST, and SM muscles from the Bísaro pig breed. Effects of diets with olive cake.

Fatty Acids	Diets					Muscles			SEM	*p*-Value		
	CD	COC	TPOC	EOC	EOC-OO	BF	ST	SM		Diets	Muscles	Diets × Muscles
C10:0	0.05 ^ab^	0.06 ^a^	0.06 ^a^	0.04 ^b^	0.04 ^b^	0.06	0.04	0.05	0.003	0.046	0.059	0.636
C14:0	1.13	1.14	1.11	1.08	1.13	1.12	1.10	1.13	0.009	0.299	0.531	0.743
C15:0	0.04	0.13	0.20	0.08	0.05	0.23	0.02	0.04	0.002	0.024	<0.001	0.008
C16:0	24.61	24.81	24.18	24.41	24.47	24.12 ^b^	24.81 ^a^	24.56 ^a^	0.080	0.078	0.001	0.659
C16:1n-7	3.28 ^b^	3.58 ^a^	3.09 ^b^	3.09 ^b^	3.08 ^b^	3.51 ^a^	2.94 ^c^	3.24 ^b^	0.057	0.002	<0.001	0.605
C17:0	0.19	0.17	0.19	0.18	0.19	0.18	0.19	0.19	0.003	0.070	0.136	0.477
C17:1n-7	0.24 ^a^	0.20 ^b^	0.21 ^b^	0.21 ^b^	0.22 ^ab^	0.21	0.21	0.22	0.004	0.014	0.767	0.696
C18:0	10.72 ^b^	10.55 ^b^	10.93 ^ab^	11.38 ^a^	10.79 ^b^	10.52 ^b^	11.51 ^a^	10.59 ^b^	0.094	0.008	<0.001	0.951
9t-C18:1	0.14	0.16	0.17	0.15	0.14	0.17 ^a^	0.12 ^b^	0.17 ^a^	0.007	0.486	0.004	0.197
C18:1n-9	51.11	50.19	49.04	50.50	50.72	50.48	50.37	50.08	0.175	<0.001	0.240	<0.001
C18:2n-6	6.23	6.81	8.18	6.50	6.85	6.69	6.88	7.18	0.147	<0.001	0.127	<0.001
C20:1n-9	0.83 ^a^	0.76 ^bc^	0.69 ^c^	0.75 ^bc^	0.78 ^ab^	0.75	0.79	0.75	0.011	0.004	0.167	0.954
C18:3n-3	0.25 ^bc^	0.26 ^bc^	0.29 ^a^	0.24 ^c^	0.27 ^b^	0.02 ^b^	0.28 ^a^	0.27 ^a^	0.005	<0.001	<0.001	0.491
C20:2n-6	0.26	0.25	0.27	0.25	0.27	0.24 ^c^	0.28 ^a^	0.26 ^b^	0.004	0.081	0.001	0.389
C20:4n-6	0.39	0.40	0.76	0.54	0.47	0.82	0.10	0.62	0.061	0.001	<0.001	<0.001
SFA	37.01	37.12	36.90	37.44	36.93	36.49 ^b^	37.91 ^a^	36.84 ^b^	0.116	0.321	<0.001	0.811
MUFA	55.69	54.96	53.33	54.79	55.00	55.25	54.45	54.57	0.180	<0.001	0.002	<0.001
PUFA	7.31	7.91	9.77	7.77	8.06	8.26	7.64	8.59	0.200	<0.001	0.007	<0.001
PUFA/SFA	0.20	0.21	0.27	0.21	0.22	0.23	0.20	0.23	0.006	<0.001	0.002	<0.001
n-6/n-3	21.21	20.50	21.88	20.98	20.78	21.00	21.44	20.77	0.257	0.521	0.566	0.309
IA index	0.46	0.47	0.45	0.46	0.46	0.45 ^b^	0.47 ^a^	0.46 ^b^	0.002	0.344	0.001	0.725
IT index	1.13	1.13	1.11	1.15	1.12	1.09 ^b^	1.17 ^a^	1.11 ^b^	0.006	0.199	<0.001	0.665
h/H	2.26	2.23	2.31	2.27	2.28	2.31	2.23	2.27	0.011	0.119	0.007	0.674

SFA—Saturated Fatty Acid; MUFA—Monounsaturated Fatty Acid; PUFA—Polyunsaturated Fatty Acid; n-6/n-3 (∑ omega-6) (∑ omega-3). IA—Index of Atherogenicity; IT—Index of Thrombogenicity; h/H—Hypocholesterolemic/hypercholesterolemic Index. CD—Control Diet; COC—Control Diet + 10% Crude Olive Cake; TPOC—Control Diet + 10% Two-Phase Olive Cake; EOC—Control Diet + 10% Exhausted Olive Cake; EOC-OO—Control Diet + 10% Exhausted Olive Cake + 1% Olive Oil; BF—*Biceps Femoris*; ST—*Semitendinosus*; SM—*Semimembranosus*; SEM—Standard Error of the Mean. Different lowercase letters (a, b, c) in the same row correspond to significant differences between diets (*p* < 0.05). ANOVA followed by a post hoc Tukey test.

**Table 5:** Fatty acid profiles (g/100 g) of BF, ST, and SM muscles from the Bísaro pig breed. Effects of diets with olive cake.

	Muscles	Diets					SEM	*p*-Value
		CD	COC	TPOC	EOC	EOC-OO		
C15:0	BF	0.06 ^b^	0.30 ^ab^	0.53 ^a^	0.19 ^b^	0.08 ^b^	0.108	0.046
	ST	0.01	0.02	0.03	0.01	0.02	0.008	0.722
	SM	0.03	0.06	0.03	0.03	0.06	0.017	0.554
C18:1n-9	BF	52.72 ^a^	50.50 ^b^	46.89 ^c^	51.15 ^b^	51.12 ^b^	0.464	<0.001
	ST	50.60	49.75	50.20	50.84	50.48	0.335	0.236
	SM	50.01	50.31	50.02	49.50	50.54	0.339	0.303
C18:2n-6	BF	5.41 ^b^	6.37 ^b^	9.46 ^a^	5.77 ^b^	6.43 ^b^	0.382	<0.001
	ST	6.17 ^c^	7.18 ^abc^	7.46 ^a^	6.27 ^bc^	7.31 ^ab^	0.342	0.046
	SM	7.11	6.89	7.61	7.46	6.81	0.395	0.555
C20:4n-6	BF	0.43 ^b^	0.79 ^b^	1.74 ^a^	0.54 ^b^	0.61 ^b^	0.125	<0.001
	ST	0.06 ^b^	0.00 ^b^	0.00 ^b^	0.17 ^ab^	0.26 ^a^	0.066	0.050
	SM	0.67	0.42	0.55	0.92	0.52	0.128	0.122
MUFA	BF	57.36 ^a^	55.74 ^b^	51.52 ^c^	55.88 ^b^	55.75 ^b^	0.447	<0.001
	ST	55.04	54.16	54.09	54.66	54.27	0.328	0.253
	SM	54.66	55.00	54.37	53.83	54.99	0.300	0.076
PUFA	BF	6.48 ^b^	7.84 ^b^	12.21 ^a^	7.00 ^b^	7.78 ^b^	0.473	<0.001
	ST	6.84 ^c^	7.86 ^abc^	8.09 ^ab^	7.09 ^bc^	8.34 ^a^	0.345	0.032
	SM	8.60	8.04	9.01	9.22	8.07	0.528	0.425
PUFA/SFA	BF	0.18 ^b^	0.22 ^b^	0.34 ^a^	0.19 ^b^	0.21 ^b^	0.013	<0.001
	ST	0.18 ^c^	0.21 ^abc^	0.21 ^ab^	0.19 ^bc^	0.22 ^a^	0.010	0.029
	SM	0.23	0.22	0.25	0.25	0.22	0.017	0.548

SFA—Saturated Fatty Acid; MUFA—Monounsaturated Fatty Acid; PUFA—Polyunsaturated Fatty Acid; n-6/n-3 (∑ omega-6) (∑ omega-3). CD—Control Diet; COC—Control Diet + 10% Crude Olive Cake; TPOC—Control Diet + 10% Two-Phase Olive Cake; EOC—Control Diet + 10% Exhausted Olive Cake; EOC-OO—Control Diet + 10% Exhausted Olive Cake + 1% Olive Oil; BF—*Biceps Femoris*; ST—*Semitendinosus*; SM—*Semimembranosus*; SEM—Standard Error of the Mean. Different lowercase letters (a, b, c) in the same row correspond to significant differences between diets (*p* < 0.05). ANOVA followed by a post hoc Tukey test. Only variables with statistically significant interactions are presented.

## Data Availability

The original contributions presented in this study are included in the article. Further inquiries can be directed to the corresponding author.
